# Remitting Seronegative Symmetrical Synovitis With Pitting Edema in an Adolescent

**DOI:** 10.7759/cureus.53853

**Published:** 2024-02-08

**Authors:** Joana Baptista de Lima, Carla Zilhão, Sérgio Alves

**Affiliations:** 1 Pediatrics, Centro Materno Infantil do Norte Albino Aroso, Centro Hospitalar Universitário do Porto, Porto, PRT; 2 Pediatric Rheumatology, Centro Materno Infantil do Norte Albino Aroso, Centro Hospitalar Universitário do Porto, Porto, PRT

**Keywords:** rheumatoid factor, tenosynovitis, seronegative, edema, synovitis

## Abstract

Remitting seronegative symmetrical synovitis with pitting edema (RS3PE) is a rare clinical entity characterized by symmetrical tenosynovitis of both hands and ankles with pitting edema, negative rheumatoid factor (RF), absence of radiographic erosions, and excellent response to low-dose steroids. It is classically associated with elderly patients but may occur in younger patients, with only one case reported in the pediatric age. We report a case of RS3PE diagnosed in a pediatric patient.

## Introduction

Remitting seronegative symmetrical synovitis with pitting edema (RS3PE) was first described by Daniel McCarty in 1985 [1]. Although initially considered a variant of rheumatoid arthritis, further research established it as a distinct and separate syndrome. RS3PE is characterized by acute-onset symmetrical tenosynovitis of the hands and ankles accompanied by pitting edema, usually also symmetrical, with negative rheumatoid factor (RF), absence of radiographic erosions, and excellent response to low-dose steroids. It occurs mostly in the elderly population and is predominantly observed in males. There are scarce case reports of individuals younger than 60 years, but to our knowledge, only one case is reported in the pediatric age [2]. Its pathophysiology is not yet fully understood, but it has been associated with neoplasms, other rheumatic diseases, and some medications.

This paper was previously presented as a meeting abstract at the 2023 Pediatric Rheumatology Annual Meeting on September 28, 2023.

## Case presentation

A 17-year-old female, with no significant past medical history, presented with a three-month history of pain and swelling in both hands (including the wrist and proximal interphalangeal joints), feet (including the ankle), and knees. Her complaints were accompanied by morning stiffness. She denied any constitutional symptoms, chronic cough, rash, ophthalmological complaints, Raynaud’s phenomenon, diarrhea, dysuria, recent infection, or recent drug or medication intake. There was no family history of rheumatic diseases or malignancies.

**Figure 1:**
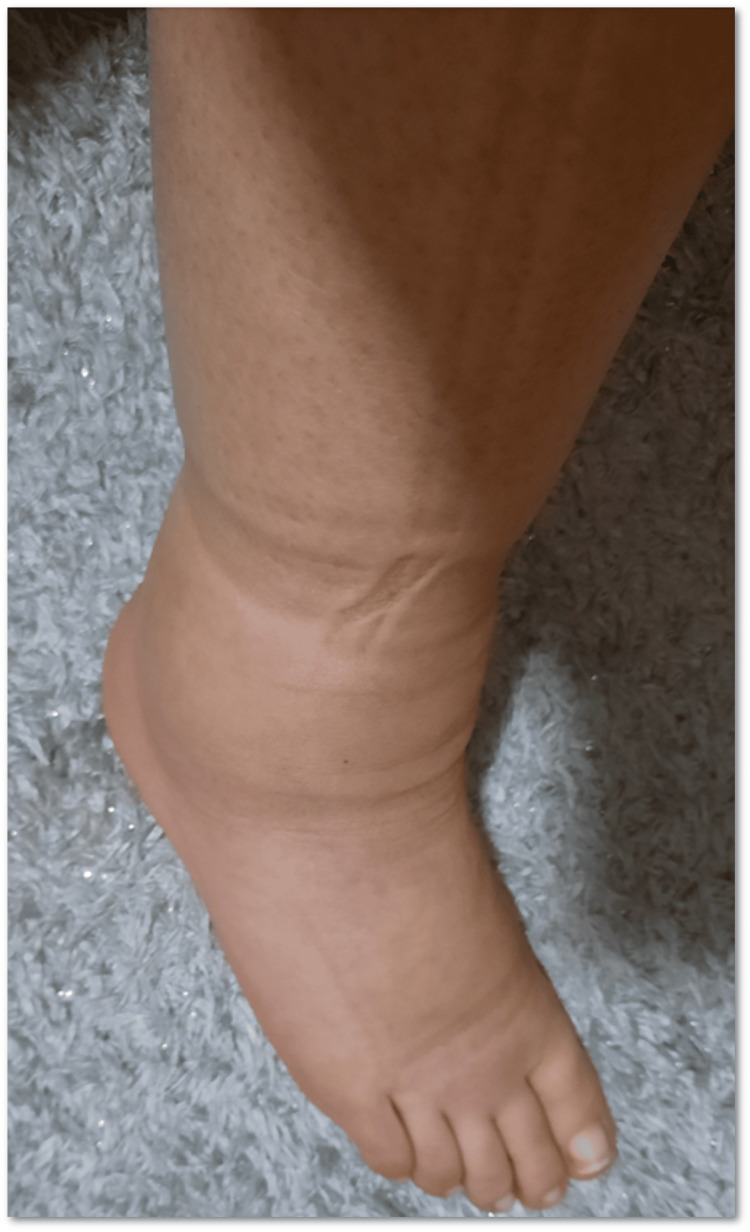
Bilateral feet edema with pitting.

**Figure 2:**
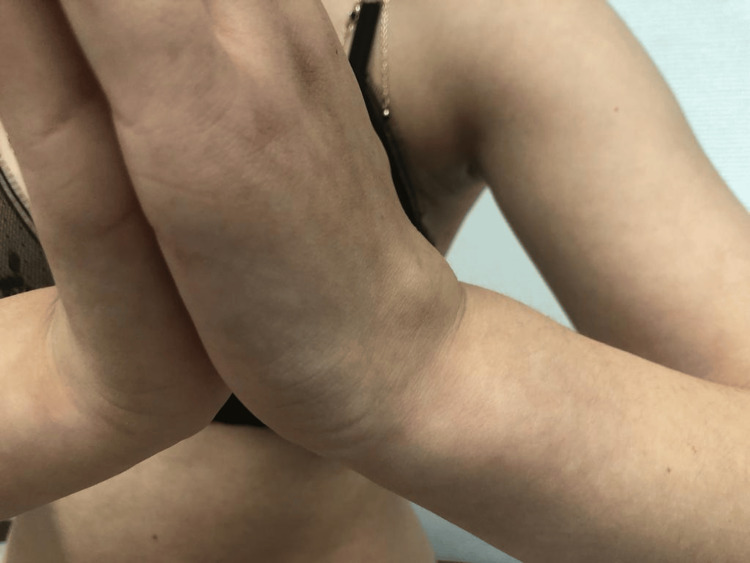
Swelling of wrists.

On examination, she presented edema of the dorsal surface of both hands extending to the wrist and prominent edema of the feet extending to the knees with pitting (Figures 1-2). 

A joint examination revealed symmetrical tenderness and swelling, with a limited range of motion of the wrists, proximal interphalangeal joints, and ankles. Her knees exhibited tenderness, warmth, and swelling, with a positive bilateral patellar tap test and a heel-to-buttock distance of 9 cm. There was no axial involvement, features of enthesitis, or ungual pitting. The presence of livedo reticularis was noted in the lower limbs. The remaining examination, including cardiovascular, respiratory, abdominal, and neurological examinations, was unremarkable.

Investigations

Laboratory investigations revealed a normal erythrocyte sedimentation rate and C-reactive protein. Albumin, liver, thyroid, renal function, and urinalysis were normal. Further investigation revealed negative anti-nuclear antibodies (ANAs), anti-double-stranded DNA (anti-ds DNA), antiphospholipid antibodies (aPL), anti-SSA and SSB, RF, anti-cyclic citrullinated peptide antibodies (anti-CCP), anti-neutrophil cytoplasmic antibodies (ANCAs), and immunoglobulin A (IgA) anti-transglutaminase. Complement (C3 and C4), immunoglobulins, and angiotensin I-converting enzyme (ACE) were within normal values. Blood tests were repeated weeks later with no change. Human leukocyte antigen-B27 (HLA-B27) was negative. Radiologic evaluation of the hands did not show erosions. Ultrasound examination of both hands and wrists showed features of extensor tenosynovitis of the hands, bilateral ankle and knee effusion with synovial hypertrophy, and diffuse soft tissue edema of the lower limbs. Interferon-gamma release assay (IGRA), chest X-ray, and fecal calprotectin were also normal.

To further exclude other possible underlying causes, the patient underwent ultrasonography of the abdomen and pelvis, peripheral blood smear, capillaroscopy, HIV test, and lower limb Doppler ultrasound, which were unremarkable. The next-generation sequencing (NGS) panel for auto-inflammatory syndromes revealed no pathological variants (Table 1).

**Table 1:** Complementary diagnostic tests. ANA, anti-nuclear antibody; anti-ds DNA, anti-double-stranded DNA; aPL, antiphospholipid antibody; anti-SSA/SSB, anti-Sjögren's Syndrome A/Sjögren's Syndrome B; RF, rheumatoid factor; anti-CCP, anti-cyclic citrullinated peptide antibody; ANCA, anti-neutrophil cytoplasmic antibody; HLA-B27, human leukocyte antigen-B27; IGRA, interferon-gamma release assay; ACE, angiotensin I-converting enzyme; NGS, next-generation sequencing

Complementary diagnostic tests	Results
Albumin, liver, thyroid, renal function, and urinalysis	Normal
ANAs, anti-ds DNA, aPL, anti-SSA/SSB, RF, anti-CCP, ANCAs, HLA-B27, IGRA, complement, immunoglobulins, fecal calprotectin, ACE	Negative
Abdominal and lower limb Doppler ultrasound, capillaroscopy, and chest X-ray	Normal
NGS panel for auto-inflammatory syndromes	No pathogenic variants

Treatment and follow-up

The patient was initially treated with naproxen with no improvement in joint involvement and worsening in lower limb edema. Low-dose prednisolone, at 15 mg (0.3 mg/kg/day), was initiated, resulting in a significant resolution of swelling and pain within three days, along with a reported weight loss of 8 kg. When tapering off prednisolone, symptoms partially returned, especially lower limb edema. It was decided to initiate a conventional disease-modifying antirheumatic drug (DMARD), specifically subcutaneous methotrexate at a dose of 20 mg/week. This allowed for the gradual tapering of prednisolone, and remission was achieved after five months of treatment. As gastrointestinal intolerance remained a challenging issue, the methotrexate dose was decreased to 17.5 mg/week with subsequent relapse of joint inflammation. Prednisolone was reintroduced with clinical response. Nevertheless, as symptoms relapsed with doses lower than 10 mg, treatment with adalimumab was initiated with subsequent clinical remission.

## Discussion

As mentioned by McCarty et al. in 1985, RS3PE is a distinct rheumatic condition, with a diagnosis not always easy to establish [1]. To standardize definitions, Olivé et al. defined diagnostic criteria, which state that the age of onset must be after 50 years old, the presence of bilateral pitting edema of hands, sudden onset of polyarthritis, and seronegativity for RF [3]. Subsequently, based on a systematic review, Karmacharya et al. suggested the following criteria for diagnosis: (1) abrupt onset, (2) marked pitting edema of mostly hands (and/or feet), (3) age of onset ≥60 years, (4) good response to short course of medium-dose steroids (10-20 mg), (4) seronegativity for RF and anti-CCP, and (5) absence of radiographic joint erosions [4]. Apart from the age, all the other diagnostic criteria were met in our patient. Moreover, five years after McCarty et al.'s description, Sattar reported three cases of RS3PE in young adults [5]. Reviewing the literature, this syndrome is extremely rare in individuals under the age of 30, and to our knowledge, there is only one case reported in the pediatric age, involving a 12-year-old girl [2].

The truly striking feature of this case is the association of pitting edema and hand tenosynovitis, making it very suggestive of RS3PE syndrome. Its pathophysiology is not well understood, but some studies suggest the role of vascular endothelial growth factor (VEGF) as a major contributor to polyarthritis and edema due to increasing vascular permeability [6]. An association with HLA-B7 and A2 has been described, but their exact role remains uncertain [7].

Due to the lack of clear diagnostic criteria and the fact that it is extremely rare in young patients, a thorough differential diagnosis must be undertaken. In this case, with the presence of arthritis and tenosynovitis, polyarticular seronegative arthritis or spondyloarthropathy could be considered among the initial possibilities. However, the remarkable presence of pitting edema is not a feature of these conditions, and when it occurs, it usually manifests as unilateral swelling of the lower limbs. It is also possible to consider this entity not as a disease per se but as a manifestation of these inflammatory arthropathies.

Systemic connectivopathies, such as systemic lupus erythematosus, could potentially explain the clinical findings, considering the possibility of lupus nephritis with nephrotic proteinuria. However, the patient's clinical features and laboratory work-up do not align with this clinical scenario. The foot edema could also be explained by primary lymphedema praecox, but it would not explain the other complaints that our patient displayed.

An association of RS3PE with tuberculosis and sarcoidosis has been reported. Nevertheless, the absence of other specific symptoms, a normal chest X-ray and ACE, and negative IGRA made this association unlikely.

Blau Syndrome is an autoinflammatory disease that manifests as polyarticular boggy synovitis and tenosynovitis, typically affecting peripheral joints, mainly wrists, knees, ankles, and proximal interphalangeal (PIP) joints of the hands. However, it generally presents as a triad of granulomatous dermatitis, arthritis, and uveitis, which was not the case for our patient. Additionally, the NGS panel for autoinflammatory syndromes did not find any pathological variant.

In elderly patients, RS3PE has been associated with malignancy, in parallel with other systemic rheumatic diseases such as inflammatory myopathies, and paraneoplastic syndromes are extremely rare in pediatrics. However, in this case, even in the absence of constitutional symptoms, given the unusual presentation, the patient underwent ultrasonography of the abdomen and pelvis and an X-ray.

Adding to the highly suggestive manifestations of the disease, the dramatic response to low-dose steroids makes RS3PE syndrome the most probable diagnosis in this case.

Nonresponse to nonsteroidal anti-inflammatory drug (NSAID) therapy is also common in this syndrome, as occurred in this patient. Due to the difficulty in tapering off prednisolone in our patient, methotrexate was started, and subsequently, a tumor necrosis factor-alpha inhibitor was needed to induce remission. According to the literature, DMARDs may be required to avoid prolonged use of steroids in about 15% of patients, and they have been effective [8].

## Conclusions

In conclusion, although RS3PE is mostly seen in elderly patients, it can occur in younger age groups, including pediatric patients, as reported in our case. This highlights the importance of reconsidering diagnostic criteria as multiple cases of younger patients have been reported in the literature. As it is an uncommon syndrome in pediatric care, it may be overlooked in pediatric rheumatology and should be considered in a typical clinical scenario.
